# *Streptococcus canis* prevalence on the normal and abnormal ocular surface of dogs referred for ophthalmic disease in Canada

**DOI:** 10.1186/s13028-023-00677-y

**Published:** 2023-04-28

**Authors:** Allyssa Cloet, Arthur Nery da Silva, Fernanda Luiza Facioli, Shayna Levitt, Lynne Sheila Sandmeyer, Matheus de Oliveira Costa, Marina Laetitia Leis

**Affiliations:** 1 Small Animal Clinical Sciences, Western College of Veterinary Medicine, Saskatoon, Canada; 2 Large Animal Clinical Sciences, Western College of Veterinary Medicine, Saskatoon, Canada; 3 Population Clinical Sciences, Faculty of Veterinary Medicine, Utrecht University, Utrecht, The Netherlands

**Keywords:** Canine, Epidemiology, Gram-positive cocci, Ocular disease, PCR

## Abstract

*Streptococcus canis* is a beta-haemolytic, Gram-positive cocci commonly identified on the canine ocular surface under both healthy and diseased conditions. The objective of the study was to determine the prevalence of *S. canis* on the normal and abnormal ocular surface of a canine ophthalmology referral population in Canada, and to investigate potential clinical aspects that may be associated with its presence. Included were 59 dogs (118 eyes) with unilateral or bilateral ocular disease diagnosed at the time of conjunctival sampling. A real-time PCR specific for *S. canis* was standardized for use with conjunctival swabs. Total DNA was extracted from 118 samples and used as template for the diagnostic assay. Samples were considered positive if amplification was detected and dissociation temperature matched a positive control. Signalment and other clinical data were also collected at the time of sampling. Of the 118 eyes sampled, 8 tested positive for *S. canis* (6.8%). No association between the detection of *S. canis* and breed, cephalic conformation, sex, age, use of ophthalmic antibiotics or other topical medications, ophthalmic diagnosis, use of systemic antibiotics or other systemic medications, or systemic diagnosis was identified. In conclusion, *S. canis* may be present on the ocular surface of dogs at a higher rate than previously reported. It is suggested that this may be linked to the use of PCR for pathogen detection instead of culture.

**Supplementary Information:**

The online version contains supplementary material available at 10.1186/s13028-023-00677-y.

## Findings

The canine ocular surface is home to a variety of microbes that, given the right circumstances, may opportunistically lead to infection. Bacterial communities that are present on a healthy corneal surface may secondarily invade corneal wounds and lead to infection once the corneal epithelial barrier is broken [1]. Bacterial keratitis in dogs is painful and can lead to partial or complete vision loss and, in severe cases, result in enucleation of the globe. Timely and targeted therapy are key in the stabilization of the corneal infection and the prevention of sequelae such as progressive keratomalacia, corneal perforation, and scarring [2]. Medical therapy may include the administration of topical non-steroidal anti-inflammatories, cycloplegics, anti-collagenolytics, and antimicrobials. The identification of a bacterial etiology is helpful to the clinician when selecting an antibiotic and aids in the judicious use of these medications.

In recent years, the role of *Streptococcus canis* in canine ulcerative corneal disease has garnered increasing attention [1, 3–5]. *S. canis* is a Lancefield group G (pyogenic) beta-haemolytic Gram-positive coccus commonly identified in ocular samples of healthy and diseased dogs [3, 6, 7]. It has been associated with otitis externa, genitourinary tract infections, pneumonia, endocarditis, arthritis, necrotizing fasciitis, septicemia, and streptococcal toxic shock syndrome in dogs [8, 9]. It has been suggested that *S. canis* isolates adapted to the corneal surface may be linked to conjunctival graft failure in dogs due to their ability to metabolize fructose which is widely available in the corneal stroma [5]. Finally, *S. canis* also has the potential to cause zoonotic disease [4, 10].

While these reports illustrate the increasing importance of this bacterium to companion animal and human health, there is a gap in the literature regarding the prevalence of *S. canis* colonization in dogs with and without ocular disease. Therefore, this study aimed to determine the prevalence of *S. canis* on the normal and abnormal ocular surface of a canine ophthalmology referral population in Canada, and to investigate potential clinical aspects that may be associated with its presence.

Canine patients presenting to a veterinary ophthalmologist at the Veterinary Medical Centre, University of Saskatchewan, Canada were recruited for the study and the following information from each case was captured: breed, sex, age, ophthalmic diagnosis, concurrent systemic disease, topical and systemic medications being administered, and topical and systemic antimicrobials being administered. The study was approved by the University of Saskatchewan’s Animal Research Ethics Board (Animal use protocol 20,180,023) and adhered to the Guidelines for Ethical Research in Veterinary Ophthalmology (GERVO).

A total of 59 dogs were included in the study, resulting in 118 samples collected (one per eye) from May 25, 2022, to June 30, 2022. Dry rayon swabs (n = 1/eye, Purflock ultra standard swab, Puritan, Guilford, ME) were used to sample the conjunctiva in the ventromedial fornix. Topical anesthetic was not applied prior to sample collection which was performed by pressing and rotating the swab between the palpebral third eyelid conjunctiva and the palpebral conjunctiva. Immediately following collection, swabs were stored in 4 °C until all collections were completed (up to 2 h) and then transferred to -80 °C until processing. All animals underwent Schirmer’s tear testing (Schirmer Tear Test Strips), rebound tonometry (Tonovet), slit lamp biomicroscopy (Kowa SL-17 Portable Slit Lamp), and indirect ophthalmoscopy (Heine Omega 500) by a veterinary ophthalmologist to determine the ocular diagnosis. Fluorescein staining was performed at the discretion of the ophthalmologist.

Total DNA was extracted from each swab using a commercial kit following the manufacturer’s guidelines (QIAmp Power Fecal Pro, Qiagen). Blank extraction controls were included with each extraction batch. Extracted DNA was then used as template for polymerase chain reaction (PCR) detection of *S. canis*.

A SYBR green real-time PCR assay was standardized using a previously published set of primers targeting the *sod*A_*int*_ gene, resulting in an amplicon of 363 base pairs [8]. A detailed description of this assay is provided in Additional file 1. Duplicates where results differed by more than 1 were repeated. PCR reactions with a fluorescence signal below 35 and a dissociation temperature that matched the positive control were considered positive. To verify assay detection limits, serial dilutions of a DNA template containing the target gene were prepared and assayed using the parameters described above.

Binary logistic regression was used to evaluate associations between a positive PCR result and breed, breed size (large *versus* small), sex, age, age group (age in years grouped as < 5 years *versus* > 5 years), cephalic conformation (brachycephalic or not), topical antibiotics, other topical medications, systemic antibiotics, other systemic medications, systemic diagnosis, any ophthalmic diagnosis, and the presence of ocular surface disease. SPSS (v23, IBM, Waltham, USA) was used for statistical testing and significance was set at 0.05.

The studied population included 59 dogs represented by castrated males (n = 19), spayed females (n = 30), intact males (n = 5) and intact females (n = 4). Standard Poodle (n = 5), Chihuahua (n = 4), Pug (n = 3) and Labradoodle (n = 3) were the only breeds with more than 2 animals included. Average age was 7.4 years (ranging from 3 months to 16 years). Most eyes (62%, 73/118) were receiving topical ophthalmic medications at the time of sampling. The majority (84%, 99/118) of eyes were not receiving topical ophthalmic antibiotics. Ciprofloxacin was used in 8% (10/118), polymyxin-bacitracin in 3% (4/118), and moxifloxacin and tobramycin in 1.7% (2/118) of the eyes included in this study. 20% (12/59) of dogs were receiving systemic medication. A total of 97% (57/59) of dogs were not receiving systemic antibiotics, while one was receiving metronidazole and one was receiving amoxicillin and clavulanic acid. Diagnoses were varied ranging from ocular surface disease to retinal disease (Table 1 and Additional file 2).

**Table 1:** **–** Data associated with cases positive for *Streptococcus canis* [OD; right eye, OS; left eye, QID; four times daily, CM; castrated male, SF; spayed female, F; female]

ID	Eye	Breed	Sex	Age (years)	Topical ophthalmic antibiotics	Topical ophthalmic medications (other)	Ophthalmic diagnosis	Systemic antibiotics	Systemic medications	Systemic diagnosis	PCR *S. canis*
2	OS	Standard Poodle	CM	1	Ciprofloxacin 4-6X daily OS	Diclofenac, atropine, serum	Healed corneal ulcer OS	none	none	none	Positive
16	OS	Australian Terrier	SF	7	none	Diclofenac	Cataract OS	none	Metronidazole, insulin	Diabetes mellitus, systemic hypertension	Positive
20	OS	Crossbreed	CM	2	none	Diclofenac	Cataract OS	none	none	none	Positive
23	OD	Standard Poodle	CM	12	none	Dexamethasone, hypertonic saline, cyclosporin A 0.2%	Entropion OD, corneal endothelial degeneration OD, follicular conjunctivitis OD, dry eye OD	none	none	none	Positive
37	OD	Chihuahua	CM	7	Ciprofloxacin QID OD	Prednisolone acetate, diclofenac, dozolamide/timolol, travoprost	Pseudophakia OD, secondary glaucoma (controlled), corneal dystrophy OD	none	none	none	Positive
39	OD	Siberian Husky Crossbreed	SF	12	none	Cyclosporin A 2%	Pannus OD, eyelid mass OD	none	Cyclosporine, levothyroxine sodium	Systemic Lupus Erythematosis, hypothyroidism	Positive
50	OS	Chihuahua	SF	8	none	Dorzolamide/timolol	Corneal endothelial degeneration OS	none	none	none	Positive
59	OS	Beagle	F	6	none	none	Iridal hyperpigmentation OS	none	none	none	Positive

A total of 6.8% of the samples (n = 8) tested positive for *S. canis* and all originated from different dogs (Table 1). The analytical range of the real-time PCR assay described here was 10^9^ to 10^2^ gene copies/µL (data not shown). Two *S. canis*-positive dogs were receiving topical ciprofloxacin at the time of sampling and none of the animals were receiving systemic antibiotics. The Standard Poodle (n = 2) and Chihuahua (n = 2) breeds accounted for 4/8 positive samples. No correlation between a positive PCR result and breed (P = 0.99), breed size (P = 0.454), cephalic conformation (P = 0.627), sex (P = 0.475), age (P = 0.263) or age group (P *=* 0.835), receipt of topical antibiotic (P = 0.108), other topical medication (P = 0.084), systemic antibiotic (P = 0.349), other systemic medication (P = 0.442), systemic diagnosis (P = 0.815), ophthalmic diagnosis (P = 0.247), or the presence of ocular surface disease (P = 0.913) was found.

*Streptococcus* spp. have been suggested to be a normal part of the ocular microbiota of dogs for decades now, based on both culture dependent and culture independent techniques [7, 11, 12]. However, studies reporting the prevalence of detection of streptococci from samples collected of diseased eyes were largely based on culture, which do not always allow for isolate speciation. As a result, the data available often mentions beta-haemolytic streptococci or *Streptococcus* spp. [12–15]. Furthermore, bacterial culture requires viable cells to be present at the time of seeding agar plates, which may result in an artificial under (or over) estimation of isolates depending on multiple factors associated with sample collection, handling, and bacterial growth conditions. For example, of the samples submitted from the ocular surface of dogs, the negative culture rate ranged from 20 to 55% [12–16]. In these studies, streptococci were cultured from the eye in 31% of isolates obtained from dogs in Australia [13], 7–17% of those in the USA (5–12% were *S. canis*) [15, 16], 8% in China (5% were *S. canis*) [12] and 7% of dogs with corneal ulcers in Taiwan [14]. If one were to take into account the negative culture rate and include all eyes swabbed in these studies, these percentages would be lower. In contrast to these studies, we detected *S. canis* in 6.8% of the *total* sample number tested and despite using a species-specific test. It is important to recognize that the ocular surface microbiota is dynamic, and likely changes in composition over time which may have an influence on the DNA load of *S. canis* resulting in a negative PCR test. This may explain why both eyes of the same dog never tested positive for the bacterium. Our data supports the already established knowledge that molecular diagnostic tests offer increased sensitivity (and specificity) for bacterial detection over classic microbiological tests. It is therefore suggested that the development of molecular diagnostic tests for ophthalmic infectious diseases should be prioritized rather than relying solely on culture, as it may aid practitioners in elucidating the etiology of infections.

We recognize that the limited number of positive samples may have hindered our ability to identify any associations between a positive PCR test and clinical factors linked to the presence of *S. canis*. Previous studies have suggested that ocular disease caused by *S. canis* may be linked to cephalic conformation as the bacterium is highly prevalent in brachycephalic breeds, particularly French Bulldogs [5, 17]. These dogs have compromised corneal health as a result of shallow orbits, prominent globes, and other breed-specific characteristics [18, 19]. While cephalic conformation could enable microenvironments for niche-specific bacteria (such as *S. canis*) to proliferate, our analyses did not identify such a relationship. A larger sample size is needed to definitively rule out any association. Including dogs from a general practice setting rather than from a strictly referral population may also translate to more generalizable results.

A previous study investigating the relevance of *S. canis* to ulcerative keratitis highlighted the potential association between strains from the MLST clonal-complex 13 and bacterial keratitis in dogs [17]. Experimentally, only *Staphyloccoccus aureus, Streptococcus pneumoniae* and *Pseudomonas aeruginosa* have been shown to cause keratitis in rabbits, mice, and guinea pigs to date [20]. However, these models were largely based on invasive techniques, inducing disease either after intracorneal injection or surgical corneal scarification. It is likely that *S. canis* is capable of invading damaged corneal epithelium based on virulence factors identified by whole genome sequencing [17]. Furthermore, it was reported that *S. canis* isolated from ulcerated corneas that failed to heal following conjunctival grafting were able to more efficiently metabolise fructose than other isolates [5]. This was suggested as a potential primary pathogenic mechanism, but still requires further evidence for confirmation.

Overall, the data presented here demonstrates that the prevalence of *S. canis* on the healthy and diseased ocular surface in a canine referral population presenting to the veterinary ophthalmologist may be higher than expected based on previous studies that used bacterial culture. Its presence and role in ulcerative keratitis and ocular surface disease is a growing concern for veterinary ophthalmologists. Future studies should focus on understanding markers of virulence to detect pathogenic strains and prevent potential detrimental outcomes.

## Electronic supplementary material

Below is the link to the electronic supplementary material.

**Additional file 1** – Supplementary methods description including PCR assay.

**Additional file 2** – All dogs included in the study regardless of PCR result.

## Data Availability

All data generated or analysed during this study are included in this article [and its supplementary information files].
