# Supplementary material for: Streptococcus canis prevalence on the normal and abnormal ocular surface of dogs referred for ophthalmic disease in Canada
Source: Acta Vet Scand. 2023 Apr 28;65:16. doi: 10.1186/s13028-023-00677-y (PMC10148516; doi:10.1186/s13028-023-00677-y)
Supplement: Supplementary file 1 — Additional file 1 – Supplementary methods description including PCR assay. [file 13028_2023_677_MOESM1_ESM.docx]

Additional file 1 – Supplementary methods description including PCR assay

Primer used were (canis-sod-I: 5'- AGAATTATTGGCAGATGTCACTA-3′; and canis-sod-ii: 5'- TTTCAAGTTGTCCTTCCTTATTG-3'). These primers were blasted against the nucleotide database of the National Center for Biotechnology Information (NCBI) to confirm specificity, and an optimal annealing temperature was identified. Specificity was evaluated using DNA from other Streptococci (*S. equi*, *S. suis, S. dysgalactiae*). Assays were performed on a Bio-Rad CFX thermocycler in reactions containing 1× SYBR Green Supermix (Bio-Rad Laboratories), 400 nM each primer and 2 μL of template DNA, in a final volume of 25 μL. All qPCR reactions were run on a plate containing a no-template control, a positive control (pure *Streptococcus canis* DNA from a clinical isolate profiled by culture and matrix-assisted laser desorption/ionization time of flight and whole genome sequencing), and extraction controls. All reactions were performed in duplicates. Thermocycling parameters included an initial denaturation (95°C for 3 min.), followed by 40 cycles of 95°C for 15 sec., 60°C for 15 sec., 72°C for 15 sec., and a final extension at 72°C for 5 min. A dissociation curve was then performed for 80 cycles at 0.5°C increments from 55°C to 95°C. Fluorescence was measured every cycle at the end of the annealing step and continuously during the dissociation curve data collection. Real-time PCR data was analyzed using iQx Optical System Software (Bio-Rad Laboratories).
